# Correlation between markers of bone metabolism and vitamin D levels in patients with monoclonal gammopathy of undetermined significance (MGUS)

**DOI:** 10.1038/s41408-017-0015-x

**Published:** 2017-12-14

**Authors:** Brea Lipe, Suman Kambhampati, Peter Van Veldhuizen, Abdulraheem Yacoub, Omar Aljitawi, Joseph Mikhael

**Affiliations:** 1 University of Rochester Medical Center, 601 Elmwood Ave Box 704, Rochester, NY 14642 USA; 2 Kansas City VA Medical Center, 4801 Linwood Blvd, Kansas City, MO 64128 USA; 3 University of Kansas Medical Center, 2650 Shawnee Mission Pkwy, Westwood, KS 66205 USA; 4 Mayo Clinic Arizona, 5777 East Mayo Boulevard, Phoenix, AZ 85054 USA

Multiple myeloma (MM) lies along a spectrum of plasma cell disorders and represents the incurable malignant phase of disease. MM is felt to be uniformly preceded by an asymptomatic clonal disease called monoclonal gammopathy of undetermined significance (MGUS) that carries a lifelong risk of disease progression^1–3^. While we are currently unable to predict which patients with MGUS will develop MM, the Mayo group has devised a risk stratification system to help in predicting patients with a higher risk of disease progression^4^. Smoldering multiple myeloma (SMM) is distinguished from MGUS by a higher burden of plasma cells and an increased risk of progression to MM^5^. There is currently no treatment for MGUS or SMM, and no treatment has been proven to reduce the risk of disease progression, though SMM remains an area of active research.

Alterations in bone metabolism, mediated in part through upregulation of receptor activator of nuclear factor κβ (RANKL) and the decoy receptor osteoprotegrin (OPG), via effects on osteoblasts and osteoclastogenesis are common to both MM and MGUS and may play a role in disease progression^6^. While patients with MGUS and SMM are generally felt to be asymptomatic, these patients do have an increased risk of bone fracture, linked to decreased bone strength and altered bone architecture^7–9^. Vitamin D is a key regulator of bone metabolism associated with progressive disease in several malignancies, including MM^10–12^. Dysregulation of RANKL and OPG correlates with disease activity and decreased survival, and has also been linked to altered bone metabolism in MGUS^13^. Based on the abnormal bone metabolism observed in patients with MGUS and the central role for vitamin D in bone metabolism, we hypothesized that abnormal bone metabolism would correlate with disease risk in MGUS and that vitamin D supplementation would improve bone metabolism and markers of disease. We investigated this hypothesis by correlating the vitamin D level with the risk category of MGUS and markers of bone metabolism, RANKL and OPG. We further evaluated the impact of vitamin D supplementation on RANKL and OPG levels, as well as markers of disease activity, including serum protein electrophoresis (SPEP) and free light chains (FLCs).

In this open-label trial, 50 patients with MGUS or SMM per the International Myeloma Working Group criteria^3^ who were older than 18 years of age were recruited to this study from a plasma cell dyscrasia clinic between June 2012 and November 2014. Patients with a history of osteoporosis, or other bone diseases, bisphosphonate usage within 1 year or glucocorticoid use within 3 months, pregnant patients, patients on bile acid sequestrants, anticonvulsive, or antiretroviral therapy, and patients with a history of malignancy other than in situ cancers within 5 years were excluded. Patients were stratified into two groups based on risk of disease progression, those with 0 or 1 risk factor for progression (low risk or intermediate-1 risk) vs. those with 2 or 3 risk factors (intermediate-2 or high risk), or SMM. Per the Mayo risk stratification system, possible risk factors include a non-IgG isotype, an IgG paraprotein level >1.5 g/dL, and an abnormal FLC ratio^4^. Ethical approval for this study was granted by the University of Kansas Ethics Committee and all patients provided informed consent.

Only patients with 25 hydroxyvitamin D (25(OH)D) < 20 ng/mL were asked to take cholecalciferol 6000 IU daily for 8 weeks followed by 2000 IU daily. Patients purchased their own cholecalciferol from a local pharmacy and were asked to complete a medication log documenting daily compliance. Patients were followed for 12 weeks.

Blood samples were collected as fasting morning labs at baseline and at 12 weeks after the initial blood sample. Full blood count, creatinine, serum paraprotein, serum FLCs, electrophoresis with immunofixation, and 25(OH)D levels were determined at each visit. Additional blood was collected in EDTA and stored at −80 degrees for plasma analysis of sRANKL, OPG, and interleukin-6 (IL-6). Total sRANKL (BioVendor), OPG (Raybiotech), and IL-6 (Raybiotech) levels were determined using enzyme-linked immunosorbent assay kits in duplicate with the lower limits of detection at 25, 1, and 0.7 pg/mL, respectively. All assays were calibrated using standard curves following the manufacturer’s instructions.

Patients were grouped according to the risk group of MGUS or SMM, and further analyzed as vitamin D-deficient vs. replete. The study planned to enroll 50 patients, 30 with low risk and 20 with low-risk MGUS to provide ~80% power to detect a 0.8 s.d. of the RANKL/OPG ratio between low- and high-risk patients for a two-sized, two-sample *t*-test with a type I error rate of 0.05 (nQuery Advisor 7.0, 1995–2007). Group values are expressed as the mean±SEM. Group comparisons were made using the unpaired Student’s *t*-test. Data within groups, pre vs. post-therapy were compared using paired Student’s *t*-test. Statistical significance was assigned as a *P* value < 0.05.

In all, 50 patients were enrolled into the study (patient characteristics Supplementary Table 1). A total of 31 patients completed the study, 5 patients were lost to follow-up prior to the initial lab draw and 14 after. There were no adverse events related to vitamin D administration.

The characteristics of low- vs. high-risk group MGUS patients, patients with low or replete vitamin D, and deficient patients prior to and after 12 weeks of supplementation are presented in Table 1. At baseline, 15 patients had MGUS with 0 risk factors, 12 with 1 risk factor, 9 with 2 risk factors, and 9 patients had SMM. Of the 31 patients completing the study, 3 patients had 0 risk factors, 7 had 1 risk factor, 9 had 2 risk factors, and 7 had SMM. At the end of 12 weeks, 2 patients who maintained 25(OH)D > 20 ng/mL went from 2 to 1 risk factor and 1 patient who developed vitamin D deficiency gained a risk factor for MGUS at 12 weeks.

**Table 1:** Characteristics by MGUS risk, vitamin D status, and impact of cholecalciferol supplementation

**Baseline characteristics of all patients**, ***N*** = **45**							**Patients with 25(OH)D < 20 ng/mL who completed the study,** ***N*** = **10**		
	Low-risk MGUS (0 or 1 risk factor)*N* = 27	High-risk MGUS (2 risk factors or SMM)*N* = 18	*P* value	25(OH)D < 20 ng/mL*N* = 15	25(OH)D > 20 ng/mL*N* = 30	*P* value	Baseline	After 12 weeks’ supplement	*P* value
SPEP (g/dL)	0.51±0.31	1.015±0.59	**0.001**	0.62±0.31	0.74±0.61	0.50	0.70±0.29	0.66±0.35	0.27
iFLC (g/dL)	4.00±7.5	19.48±21.4	0.43	11.50±23.00	9.45±21.27	0.39	15.71±27.16	6.9±13.2	0.2
Ratio involved/uninvolved chain	3.25±4.55	23.67±34.29	0.14	55.56±134.05	14.47±33.28	0.19	5.38±7.99	1.14±0.66	**0.03**
25(OH)D level (ng/mL)	29.26±10.9	31.21±5.6	0.56	16.73±3.30	36.70±6.83	0.0001	16.02±3.5	25.34±10	**0.04**
On 25(OH)D baseline # (%)	15 (56)	10 (56)		8 (53%)	17 (57%)				
RANKL (pg/mL)	14 685.6±6729.1	46 037.0±9919.6	**0.04**	51 994.82±97541.71	14 453.24±19 415.21	**0.03**	63 218±107 320	45 765±81 420	**0.039**
OPG (pg/mL)	164.94	249	0.25	177.78±63.37	165.25±90.68	0.3	185.55±68.72	201.6±62.37	**0.04**
RANKL/OPG	96.92±60.26	249.54±105.3	**0.048**	260.77±448.24	102.69±122.74	**0.05**	344.24±506.67	211.7±307.8	**0.04**
IL-6 (pg/mL)	3.56	3.42	0.32	4.05±3.92	3.20±3.93	0.26	3.91±4.4	3.69±3.93	0.33

Statistically significant values are shown in bold

A total of 32 (71%) patients were either found to be vitamin D-deficient or had a history of vitamin D deficiency requiring vitamin D supplementation at baseline. Patients with vitamin D deficiency had higher levels of RANKL and the RANKL/OPG ratio than replete patients (Table 1). Seventy percent of patients had improvement in their 25(OH)D level with supplementation. After supplementation, the vitamin D-deficient patients had decreases in RANKL, RANKL/OPG ratio with an increase in the OPG levels (Table 1). A decrease in the involved FLC (iFLC), FLC ratio, and SPEP were seen after cholecalciferol supplementation, but improvements were greatest for patients who had improvements in 25(OH)D levels. After 3 months, 76% of patients with 25(OH)D > 20 ng/mL had decreases in their 25(OH)D levels, (37.58 vs. 34.81, *P* = 0.07) with an increase in the iFLC (7.84 vs. 10.75, *P* = 0.06) and RANKL (7987.5 vs. 13351.0, *P* = 0.07). Figure 1 summarizes the differences between the FLC ratio and RANKL/OPG ratio amongst the patients in our study.

**Fig. 1:**
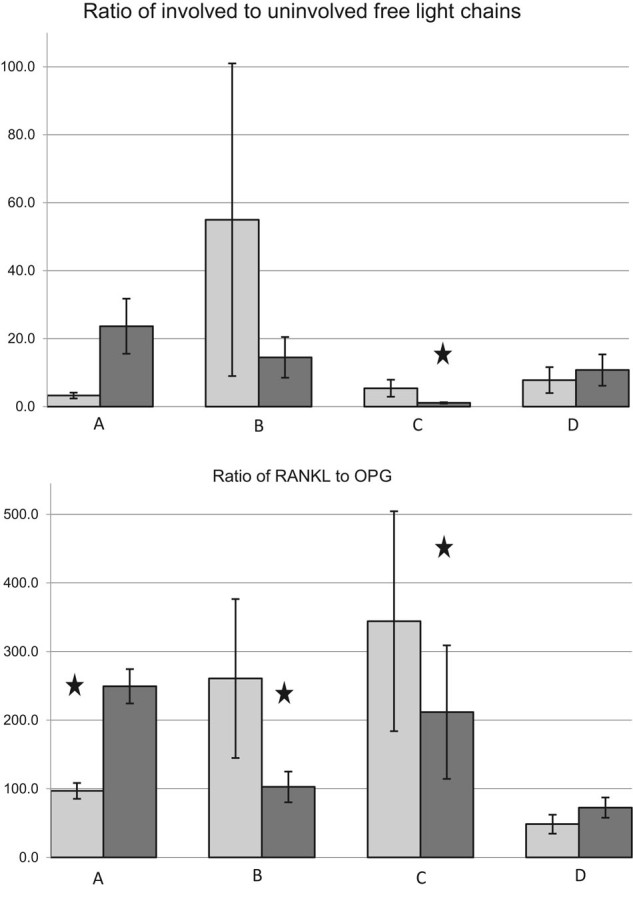
Graphic representation of FLC ratio and RANKL/OPG ratio **A** at baseline between patients with low- vs. high-risk MGUS, **B** patients with vitamin D > 20 vs. < 20 ng/mL, **C** patients with low vitamin D prior to and after 12 weeks of repletion, and **D** patients with replete vitamin D at baseline and after 12 weeks without supplementation

In this prospective study, we have demonstrated a correlation between progressive alteration in bone metabolism via RANKL and OPG with an increased risk of progression from MGUS to MM as determined by the Mayo risk stratification criteria^4^. We further demonstrate that supplementation with vitamin D can improve markers of bone metabolism, RANKL, and OPG, while reducing markers of disease activity. To our knowledge, this is the first study to correlate bone metabolism and vitamin D supplementation with a known risk for disease progression.

We further demonstrate that RANKL and RANKL/OPG are increased with high-risk MGUS and SMM vs. low risk. These data support a link between disease activity and bone metabolism for patients without clinically relevant plasma cell disorders and may provide a mechanism for the bone abnormalities and increased risk of fracture that is seen in patients with MGUS. In our study, vitamin D deficiency was independently linked to bone metabolism. Our study was confounded by the high baseline rate of vitamin D supplementation within our patient population. Based on patient history and the observed 25(OH) D levels within our study, 71% of patients were vitamin D-deficient, consistent with prior data^14^. Within our study, several patients who reported taking vitamin D supplementation (6 patients) had 25(OH)D levels < 20 ng/mL. Additionally, several patients within our study who were assigned to take cholecalciferol, failed to have restoration of their 25(OH)D levels. Despite medication logs, it is impossible to exclude poor compliance, seasonal variation, or malabsorption as a mechanism for the failure of cholecalciferol supplementation. However, the correlation between vitamin D status and bone metabolism independent of supplementation, suggests a possible difference in disease biology amongst patients whose 25(OH)D levels improve with supplementation vs. those who do not.

The iFLC, FLC ratio, and SPEP represent surrogates for disease activity in plasma cell dyscrasias, including MGUS and SMM^4^. Within our study, supplementation with cholecalciferol improved 25(OH)D levels and correlated with a decrease in markers of bone metabolism and disease activity as measured by iFLC, FLC ratio, and the SPEP. Since baseline supplementation status was not correlated with disease activity, our study suggests a direct action of cholecalciferol on disease biology that may be mediated through improvements in bone metabolism.

In our study, a large number of enrolled patients failed to complete the study. Likewise, it is impossible to determine if low 25(OH)D levels contribute to altered bone metabolism and disease progression or if alterations in disease biology lead to altered 25(OH)D levels. However, our data do suggest a role for vitamin D in plasma cell mediated bone disease and disease progression that should be further studied in a larger study across the spectrum of disease activity.

## Electronic supplementary material

Supplementary Table 1

## References

[1] Landgren O (2009). Monoclonal gammopathy of undetermined significance (MGUS) consistently precedes multiple myeloma: a prospective study. Blood.

[2] Weiss BM, Abadie J, Verma P, Howard RS, Kuehl WM (2009). A monoclonal gammopathy precedes multiple myeloma in most patients. Blood.

[3] Kyle RA (2010). Monoclonal gammopathy of undetermined significance (MGUS) and smoldering (asymptomatic) multiple myeloma: IMWG consensus perspectives risk factors for progression and guidelines for monitoring and management. Leukemia.

[4] Rajkumar SV (2005). Serum free light chain ratio is an independent risk factor for progression in monoclonal gammopathy of undetermined significance. Blood.

[5] Kyle RA (2007). Clinical course and prognosis of smoldering (asymptomatic) multiple myeloma. N. Engl. J. Med..

[6] Roodman GD (2010). Pathogenesis of myeloma bone disease. J. Cell Biochem..

[7] Kristinsson SY (2010). Monoclonal gammopathy of undetermined significance and risk of skeletal fractures: a population-based study. Blood.

[8] Melton LJ (2004). Fracture risk in monoclonal gammopathy of undetermined significance. J. Bone Miner. Res..

[9] Farr JN (2014). Altered cortical microarchitecture in patients with monoclonal gammopathy of undetermined significance. Blood.

[10] Bikle D (2009). Nonclassic actions of vitamin D. J. Clin. Endocrinol. Metab..

[11] Ng AC, Kumar SK, Rajkumar SV, Drake MT (2009). Impact of vitamin D deficiency on the clinical presentation and prognosis of patients with newly diagnosed multiple myeloma. Am. J. Hematol..

[12] Diamond T, Golombick T, Manoharan A (2010). Vitamin D status may effect the skeletal complications of multiple myeloma. Am. J. Hematol..

[13] Politou M (2004). Role of receptor activator of nuclear factor-kappa B ligand (RANKL), osteoprotegerin and macrophage protein 1-alpha (MIP-1a) in monoclonal gammopathy of undetermined significance (MGUS). Br. J. Haematol..

[14] Badros A (2008). Prevalence and significance of vitamin D deficiency in multiple myeloma patients. Br. J. Haematol..

