# Supplementary material for: Correlation between markers of bone metabolism and vitamin D levels in patients with monoclonal gammopathy of undetermined significance (MGUS)
Source: Blood Cancer J. 2017 Dec 14;7(12):646. doi: 10.1038/s41408-017-0015-x (PMC5802504; doi:10.1038/s41408-017-0015-x)
Supplement: Supplementary file 1 — Supplementary Table 1 [file 41408_2017_15_MOESM1_ESM.docx]

|  | Baseline (45 patients) | After 12 weeks (31 patients) |
| --- | --- | --- |
| Males (#, %) | 18, 40% | 14, 45% |
| Females (#,%) | 27, 60% | 17, 55% |
| Age (Mean +/- SD) | 65 +/- 12.5 years | 66 +/- 12.6 years |
| Caucasian (#, %) | 42, 93% | 28, 90% |
| African American (#, %) | 3, 7% | 3, 10% |
| MGUS 0 risks (#, %) | 15, 33% | 7, 23% |
| MGUS 1 risk (#, %) | 12, 27% | 8, 26% |
| MGUS 2 risks (#, %) | 9, 20% | 9, 29% |
| MGUS 3 risks (#, %) | 0 | 0 |
| SMM (#, %) | 9, 20% | 7, 23% |
| On vitamin D at baseline (#,%) | 34 , 76% | |
| 25(OH)D < 20ng/mL | 15, 33% | 8, 26% |
| 25(OH)D > 20ng/mL | 30, 67% | 23, 74% |

**Table 1:** Characteristics of patients enrolled at baseline and at 12 weeks of follow up.
